# Supplementary material for: Use Treadmills with Caution: Walking Energy Expenditure and Metabolic Cost Are Elevated Compared to Overground Across Multiple Speeds in Healthy Young Adults
Source: J Funct Morphol Kinesiol. 2026 May 29;11(2):220. doi: 10.3390/jfmk11020220 (PMC13302541; doi:10.3390/jfmk11020220)
Supplement: Supplementary file 1 [file jfmk-11-00220-s001.zip › jfmk-4308854-supplementary.pdf]

Supplementary Figure

Males

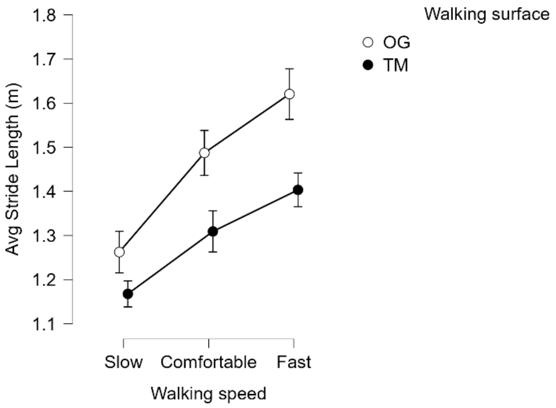

Females

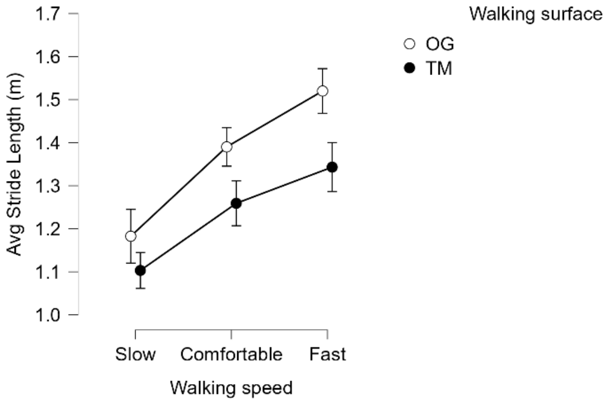

Figure S1: Line-plots of average stride length are shown for overground (OG, in open white circles) and treadmill (TM, in black closed circles). The circles denote the mean values and the vertical straight black lines across the circles are the error bars denoting the 95% CIs. The x-axis shows the walking speeds in text and the y-axis shows the gait parameter and its unit.
